# Methodology Proposal of EMG Hand Movement Classification Based on Cross Recurrence Plots

**DOI:** 10.1155/2019/6408941

**Published:** 2019-12-04

**Authors:** M. A. Aceves-Fernandez, J. M. Ramos-Arreguin, E. Gorrostieta-Hurtado, J. C. Pedraza-Ortega

**Affiliations:** Universidad Autónoma de Querétaro, Faculty of Engineering, Cerro de las Campanas S/N, Querétaro 76010, Mexico

## Abstract

Dealing with electromyography (EMG) signals is often not simple. The nature of these signals is nonstationary, noisy, and high dimensional. These EMG characteristics make their predictability even more challenging. Cross recurrence plots (CRPs) have demonstrated in many works their capability of detecting very subtle patterns in signals often buried in a noisy environment. In this contribution, fifty subjects performed ten different hand movements with each hand with the aid of electrodes placed in each arm. Furthermore, the nonlinear features of each subject's signals using cross recurrence quantification analysis (CRQA) have been performed. Also, a novel methodology is proposed using CRQA as the mainstream technique to detect and classify each of the movements presented in this study. Additional tools were presented to determine to which extent this proposed methodology is able to avoid false classifications, thus demonstrating that this methodology is feasible to classify surface EMG (SEMG) signals with good accuracy, sensitivity, and specificity. Lastly, the results were compared with traditional machine learning methods, and the advantages of using the proposed methodology above such methods are highlighted.

## 1. Introduction

Electromyography (EMG) has been investigated for many decades. The nonlinear nature of these time-series signals has been the focus of studies and the motivation of many works where many authors have developed many techniques to deal with this nonlinear nature of the signals [1, 2]. There are many hurdles when dealing with EMG signals. Some of them are as follows:

- Linear approaches are often not accurate enough when dealing with EMG signals
- There is a good probability of the existence of a signal (for example, when a movement is carried out by a person), but many existing methods have problems locating the signal itself
- The noise embedded in the signal may greatly increase the complexity of the movement classification
- Also, noise buried in the signal often makes the classification difficult
- Most machine learning methods require that a preexisting model of the signals' behavior exists and a training method must often be defined

To deal with these problems, a novel methodology is presented using feature reduction, feature selection, wavelet denoising, and cross recurrence analysis in the present work.

As it was stated before, there are many difficulties when dealing with time-series analysis, where linear approaches do not necessarily suffice to analyze some type of data.

To overcome these difficulties with highly nonlinear, nonstationary data series, the method of recurrence plots (RP) has been introduced [3, 4]. An additional quantitative analysis of recurrence plots has been developed to detect transitions in complex systems [5, 6].

RPs are graphical representations of the amount of time at which two states of a system are close to each other; this is, they exist in the same phase-space neighborhood. With these graphical representations, the dynamics of a high-dimensional system may be studied [7]. Furthermore, a new method called cross recurrence plot (CRP) has become popular recently. This has proven useful for noisy, nonstationary data, which make this tool advantageous when dealing with EMG signals. Since the information from CRP is embedded in a visual representation of the data, cross recurrence quantification analysis (CRQA) has been developed. With CRQA, the number and duration of recurrences of a nonlinear, dynamic system can be presented by examining at its phase trajectory and quantify it [8].

## 2. Background

### 2.1. EMG Signals

**Figure 1:**
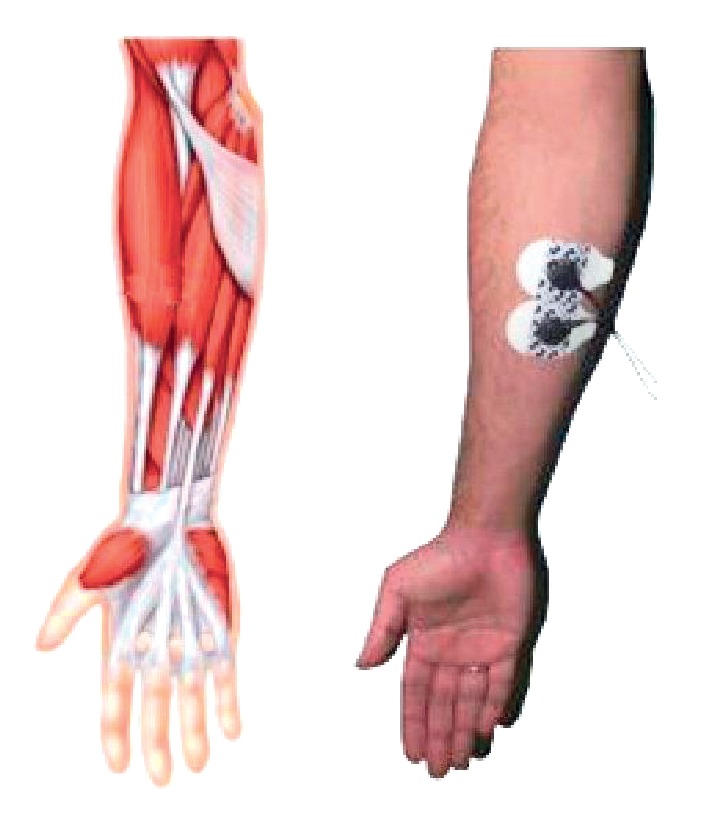
Surface electromyography example [2].

Electromyography (EMG) detects the muscle signal, which is commonly detected by electrode pairs with the purpose of extracting information [1]. EMG can be invasive and noninvasive. Generally speaking, a noninvasive technique shows a greater noise embedded in the muscle's signal. Nevertheless, in this study a noninvasive technique called SEMG (surface EMG) was used to acquire the signals, which made the classification task more challenging but represented little to no discomfort at all for all the subjects in this study. An example of the muscle and positioning of the paired electrodes for SEMG is shown in Figure 1.

**Figure 2:**
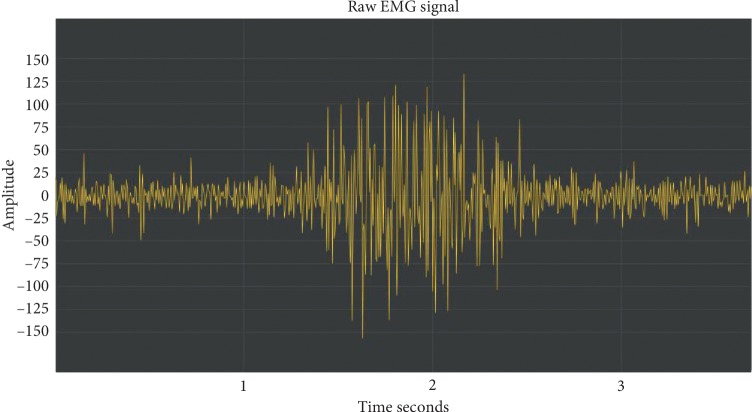
Example of a raw SEMG signal event.

The raw signal of a movement acquired using the methodology proposed in the present study can be seen on Figure 2.

### 2.2. EMG Classification

Many studies aimed to classify EMG signals using different techniques and methodologies. For instance, Sezgin [9] had successfully classified sleep apnea syndrome using wavelet and extreme learning machine, whilst Jali et al. [10] had characterized EMG signals of arm's flexion using self-organizing maps (SOM). Also, Harazika et al. [11] proposed a hybrid methodology using a canonical correlation analysis (CCA) and a classification method called KNN. Furthermore, Zhai et al. [12] showed the feasibility of classifying EMG signals using a spectrogram and principal component analysis. Gokgoz and Subasi [13] had used tree algorithms and wavelet discrete transform to classify EMG signals. Lastly, Khushaba et al. [14] had tried several pattern recognition algorithms for EMG muscular contraction.

### 2.3. Wavelet Denoising

There have been many works in which wavelet has been used to remove the noise artifacts embedded in a signal [15, 16]. For instance, El-Dahshan [17] proposed a hybrid method using Genetic Algorithms and Wavelet transform to remove the noise of electrocardiogram (ECG) signals, whilst Gao et al. [18] and Mamun et al. [19] shows the feasibility of using Wavelet for reducing noise of electroencephalogram (EEG) signals. Also, Ergen [20] had successfully used wavelet denoising in a variety of types of signals and images. Furthermore, Sen et al. [21] and Litak et al. [22] showed that it is feasible to use wavelet transfer in nonlinear systems such as cutting processes in milling. Lastly, Hussain et al. [23] was able to use wavelet analysis and an EMG signal to determine muscle contractions.

For these reasons, wavelet denoising was chosen to remove unwanted noise in the highly nonlinear, multichannel signals as wavelet provides possibilities to study both frequency and time maps on a signal simultaneously. Also, wavelet denoising is found to be efficient as many authors [15–19, 23] indicate that wavelet preserves the main signal features while reducing noise.

The properties of a family of wavelets depend upon the mother wavelet's features. A wavelet aims to decompose a signal in various parts that conforms to a basic function called “wavelet function” [19]. The influence of the selection of wavelet function and the choice of the decomposition level and the parameter will depend heavily upon the success of the filtering as shown by El-Dahshan [17].

The wavelet transform (WT) gives a decomposition of *x*(*t*) in different scales, tending to fit the scales at time locations where the wavelet best resembles *x*(*t*) [20]. This process can be reversed, thus giving the reconstruction of *x*(*t*). According to many authors [19, 20, 23], it is more convenient to define the WT only at discrete scales *a* and discrete times *b* by choosing the following set of parameters: {*aj* = 2 − *j*; *bj*, *k* = 2 − *jk*} where *j* and *k* are integers.

WT is defined as the convolution between the signal *x*(*t*) and the wavelet functions *ψ*_*a*, *b*_(*t*) (equation):

(1) $$\begin{array}{c} W_{\psi }X\left(a,b\right)=x\left(t\right)\;\left|\;\right.\psi_{a,b}\left(t\right), \end{array}$$

where *ψ*_*a*,*b*_(*t*) are shifted versions of a wavelet function *ψ*(*t*).

(2) $$\begin{array}{c} \psi_{a,b}={\left|a\right|}^{-1/2}\psi \left(\frac{t-b}{a}\right). \end{array}$$

### 2.4. Cross Recurrence Plots

Recurrence plots and cross recurrence plots have been used extensively in the literature for a variety of applications. For instance, Demos and Chaffin [24] have identified movements in musical performance using recurrence plots. Also, Popescu et al. [25] have characterized an electric fingerprint for home appliances using RPs, whilst Litak et al. [26, 27] has used RPs to describe the vibrations for milling process and Aceves [7] have found trends of Airborne Particulate Matter (PM10) over long periods of time. Furthermore, some works such as the ones carried out by Addo et al. [28] and Bastos and Caiado [29] have shown that financial applications also use successfully RPs to detect for instance measures of predictability and dependencies in stock markets.

In terms of CRP, Villamor and Rodrigo [30] had used cross recurrence quantification analysis (CRQA) to discern between novice and expert programmers by tracking eye movements, whilst Elias and Narayanan Namboothiri [8] had implemented a methodology based upon CRP to analyze vibration signals in machinery. Other works such as the ones performed by Aboofazeli and Moussavi [31] and Rashvandi and Nasrabadi [32] have been able to distinguish between different breath sounds using RQA. Furthermore, Ngamga et al. [33] have been able to identify seizures in patients suffering epilepsy, while other authors such as Goshvarpour and Goshvarpour [34] and Mazaheri et al. [35] have accurately used CRQA for biomedical applications.

Finally, Nalband et al. [36] has used RQA to characterize knee-joint disorders, whilst Silva et al. [37] used RQA to classify EMG signals for low-back pain applied to golf swings. Finally, the authors acknowledge that, in terms of related work, Ouyang et al. [38, 39] has characterized hand grasp using recurrence plots, although such authors do not use a joint space in a cross recurrence plot nor a formal methodology using data preprocessing. No other related work has been found that tackles the problems layout in the introduction section of this document. Since RP and CRP have the advantage of easily interpreting the results, as shown in this section, they have been used extensively to detect features in signals including EEG and EMG signals, and cross recurrence-based dynamics quantification of SEMG signals appears to be a promising choice.

A cross recurrence plot (CRP) is a two-dimensional figure that represents the occurrences between two different dynamic systems in an m-dimensional phase space. Cross recurrent matrix is defined by Marwan and Kurths [40] as

(3) $$\begin{array}{c} {\text{CR}}_{i,j}=\Theta \left(\varepsilon -\left\|{\overset{\to }{x}}_{i}-{\overset{\to }{y}}_{j}\right\|\right),\;i=1,\ldots ,N,\;j=1,\ldots ,M, \end{array}$$

where ${\overset{\to }{x}}_{i}$ and ${\overset{\to }{y}}_{j}$ represent the trajectories in an m-dimensional space, Θ(·) is the Heaviside function, and ‖·‖ is the Euclidean norm.

**Figure 3:**
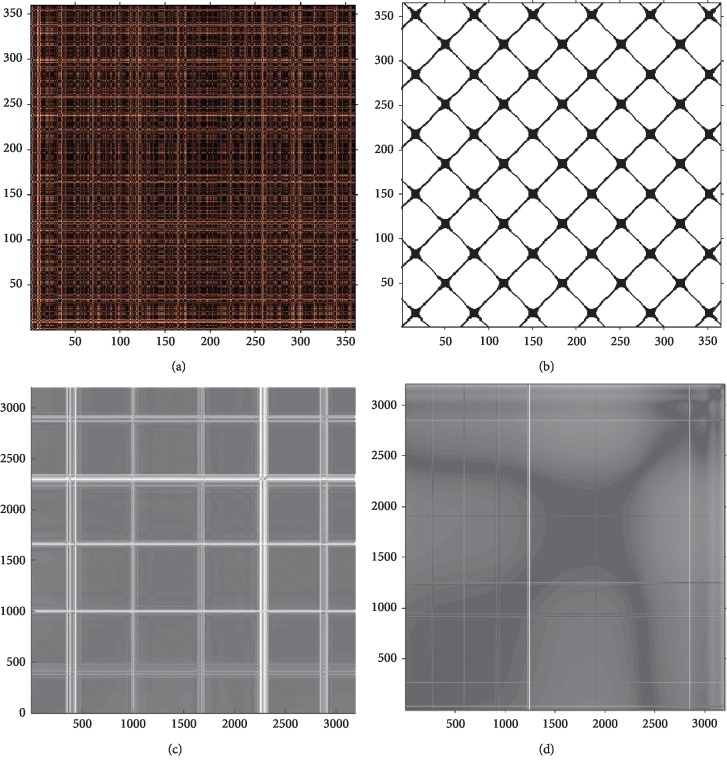
Recurrence plots using (a) a random signal, (b) a sine wave, (c) cubital wrist deviation movement subject 03 left hand, and (d) initial position (wrist in neutral) Subject 16 right hand.

Since CR*i*, *i* = 1 (*i* = 1,…, *N*) by definition, the RP has a black main diagonal line called line of identity (LOI), which occurs when the trajectory visits the same region of the phase space at different times and its length depends on the largest Lyapunov exponents. Different patterns can be observed according to the features of the signal. In Figure 3 is shown the graphical representation of different signals. For instance, Figure 3(a) shows a random signal, whilst Figure 3(b) shows a sine wave. Interestingly, Figures 3(c) and 3(d) show a randomly selected movement, subject, and hand used in this experiment which will be explained in detail on Section 3.1.

There are certain parameters, called embedding parameters, which must be calculated first according to several authors, i.e., [40–43]. These parameters are time delay, embedding dimension, norm, and recurrence threshold. The main objective of the time-delay embedding is to unfold the phase-space trajectory in a sufficiently large space, since the noise is likely to increase as the dimension increases due to the nonlinearity of the signal. In this contribution, the time delay was set to 1 using average mutual information (AMI). Having a proper time-delay setup allows to accurately reconstruct the signal to its original phase space, since having a high time-delay the chaotic attractors will fold, thus giving an incorrect phase-space reconstruction.

Also, the embedding dimension was calculated using the FNN algorithm (False Nearest Neighbors) as shown on Zou et al. [44], which in this case was calculated as *m* = 6. According to Takens' theorem [45], if the embedded dimension *m* is considerably higher than the dimension of the system, it is possible to reconstruct the original phase-space topology. Furthermore, the norm was chosen as Euclidean since it was demonstrated that it gave the most accurate results [42, 44].

Lastly, the recurrence threshold *ε* must be a trade-off between having as small as possible and also sufficiently large with some authors, i.e., [7, 46] stating that the recurrence threshold must be as large as five times the standard deviation of the observational noise, i.e., *ε* > 5*σ*. In a previous contribution [7], it was used a threshold of 6 since the noise was unknown. However, since one of the main contributions of this paper is the removal of noisy artifacts that may affect the detection of the signal, the threshold was set to a relatively small number, i.e., *ε* = 2.

Once the tuning parameters for cross recurrence plot have been defined, a quantification of the recurrence structures must be calculated. This quantification has been mentioned by several authors [4, 29, 33, 47, 48], commonly known as cross recurrence quantification analysis (CRQA). The measures considered in this contribution are Recurrence Rate, Determinism, Entropy, Laminarity, Trapping Time, and Trend.

#### 2.4.1. Recurrence Rate

The recurrence rate is a measure of recurrences, or density of recurrence points in the RP. This rate gives the mean probability of recurrences in the system [47]. The recurrence rate is given by

(4) $$\begin{array}{c} \text{RR}=\frac{1}{N^{2}}\overset{N}{\underset{i,j}{\sum }}R_{i,j}=\frac{1}{N^{2}}\overset{N}{\underset{l=1}{\sum }}lP\left(l\right). \end{array}$$

#### 2.4.2. Determinism

Determinism (DET) corresponds to the local predictability of a system. This measure ranges from 0 to 1, where numbers near 0 indicate randomness and those approaching to 1 indicate the presence of a strong signal component [3, 4]. Determinism of a system is calculated as

(5) $$\begin{array}{c} \text{DET}=\frac{\sum_{l\geq l_{\min}}lP\left(l\right)}{\sum_{i,j}R_{i,j}}. \end{array}$$

#### 2.4.3. Entropy

This measure refers to the Shannon entropy of the frequency distribution of the diagonal line lengths [49].

The entropy of a system is given by

(6) $$\begin{array}{c} \text{ENT}=-\overset{N}{\underset{l=l_{\min}}{\sum }}p\left(l\right)\log\;p\left(l\right)\text{with }p\left(l\right)=\frac{p^{\varepsilon }\left(l\right)}{\sum_{l=l_{\min}}^{N}P^{\varepsilon }\left(l\right)}. \end{array}$$

#### 2.4.4. Laminarity

Laminarity (LAM) may be defined as the frequency distribution of the lengths that form vertical lines [6]. Laminarity is also the evidence of the chaotic transitions and is related to the number of laminar phases in the system, i.e., the intermittency of a system.

(7) $$\begin{array}{c} \text{LAM}=\frac{\sum_{v=v_{\min}}^{N}v,P\left(v\right)}{\sum_{v=1}^{N}v,P\left(v\right)}. \end{array}$$

#### 2.4.5. Trapping Time

Trapping Time shows the average length of the vertical lines and is given by equation

(8) $$\begin{array}{c} \text{TT}=\frac{\sum_{v=v_{\min}}^{N}vP\left(v\right)}{\sum_{v=v_{\min}}^{N}P\left(v\right)}, \end{array}$$

where *v* is the length of the vertical lines, *v*_min_ is the shortest length that is considered a line segment, and *P*(*v*) is the distribution of the corresponding lengths. TT shows the time that the system has been trapped in the same state [50].

#### 2.4.6. Trend

The trend is a linear regression coefficient over the recurrence point density of the diagonals parallel to the Line of Identity (LOI). The trend measurement is given by

(9) $$\begin{array}{c} \text{TREND}=\frac{\sum_{i=0}^{N}\left(i-\left(N/2\right)\right)\left(RR_{i}=\left\{RR_{i}\right\}\right)}{\sum_{i-1}^{N}{\left(i=\left(N/2\right)\right)}^{2}}. \end{array}$$

## 3. Materials and Methods

### 3.1. SEMG Tests

A total of fifty subjects, with no history of muscular disorders or pain were selected. The subject's selection was chosen so that different signal profiles are used, showing that the proposed methodology is feasible regardless of the hand used in the experiment. which makes this problem far more complex in its classification.

In this context, 21 subjects were women and 29 men, between ages 18–60 as given in Table 1. All subjects gave informed consent to participate in the experimental procedure. Also, this study is based on the ethical considerations raised in the treaty of International Ethical Guidelines for Biomedical Research in Human Beings (World Health Organization, & Council for International Organizations of Medical Sciences, 2016) by the Council for International Organizations of Medical Sciences (CIOMS) in collaboration with the World Health Organization (WHO).

The muscles associated with the movements were triceps brachii, anconeus, brachioradialis, and pronator teres. There are two sensors for each muscle, paired and separated by 2 cm (roughly 0.78 in) from each other.

**Figure 4:**
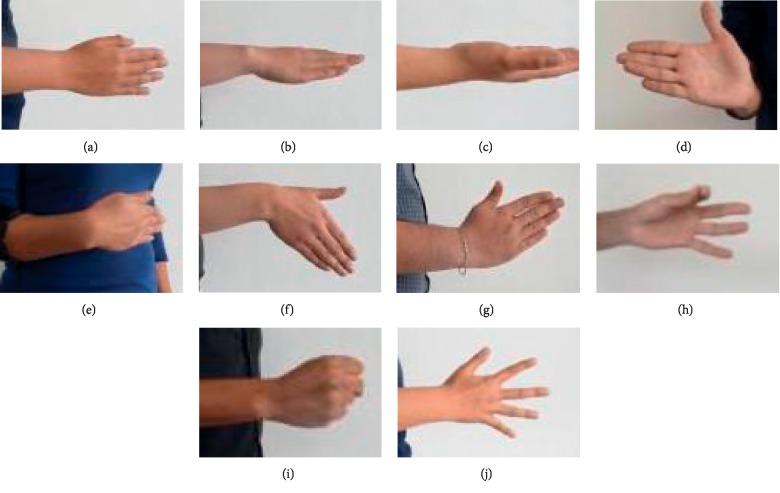
List of movements every subject performed. (a) Initial position, (b) pronation, (c) supination, (d) wrist extension, (e) wrist flexion, (f) cubital wrist deviation, (g) radial wrist deviation, (h) hand picking, (i) hand closed, (j) hand open.

The list of movements that were designed for the acquisition of the experiments presented in this study is as follows (presented in the actual order of execution):

1. Initial position (rest position): in this position, the subject keeps the elbow parallel to the ground, with his/her fingers extended, without putting much strength on them. From this posture will start each of the events of every one of the other nine movements (Figure 4(a)).
2. Pronation: starting with the arm in the initial position close to the torso with the palm perpendicular to the ground, a rotation is done so that the palm faces downwards. After this, the hand returns to the initial position (Figure 4(b)).
3. Supination: starting with the arm in the initial position, a rotation is done so that the palm faces upwards. After this event, the hand returns to the initial position (Figure 4(c)).
4. Wrist extension: starting with the arm in the initial position, a wrist movement in which the palm of the hand moves away from the body, avoiding moving the whole arm. After this event, the hand returns to the initial position (Figure 4(d)).
5. Wrist flexion: starting with the arm in the initial position, a wrist movement in which the palm of the hand moves inwards towards the body. After this event, the hand returns to the initial position (Figure 4(e)).
6. Cubital wrist deviation: starting with the arm in the initial position, without moving the elbow and arm from the 90° angle, the wrist is inclined downwards towards the floor avoiding forcing the wrist to an uncomfortable position. After this event, the hand returns to the initial position (Figure 4(f)).
7. Radial wrist deviation: starting with the arm in the initial position, without moving the elbow and arm from the 90° angle, the wrist is inclined upwards away from the floor avoiding forcing the wrist to an uncomfortable position. After this event, the hand returns to the initial position (Figure 4(g)).
8. Hand picking: starting with arm in the initial position, the tip of the index finger and thumb must come closer until they gently touch each other. After this event, the hand returns to the initial position (Figure 4(h)).
9. Hand closed: starting with arm in the initial position, without moving the elbow and the arm from the initial 90° angle, the subject must close his/her hand gently without applying excessive force to the grasp movement. After this event, the hand returns to the initial position (Figure 4(i)).
10. Hand open (finger extension): starting with arm in the initial position, without moving the elbow and the arm from the initial 90° angle, the subject is asked to gently extend his/her fingers so that there is a separation between each of the fingers without applying excessive force to this finger extension movement (Figure 4(j)).

It is important to notice that, in the movements, no extra force is required and the thumb is not forced to be in a 90° angle from the others fingers. This is advised to every subject due to the fact that what the experiment aims for is to classify casual and “natural” movements. Also, as it was explained before, every movement for each subject consists of five events evenly distributed on a timeframe of sixteen seconds.

### 3.2. Proposed Methodology

The proposed methodology consists of the following steps:

- Acquisition of the signals (all electrodes) for each movement.
- The experiment must be performed in the same order of movements always. That is (1) initial position (wrist in neutral), (2) pronation, (3) supination, (4) wrist extension, (5) wrist flexion, (6) cubital wrist deviation, (7) radial wrist deviation, (8) hand picking, (9) hand closed and (10) hand open.
- The number of the events for each movement and each hand is always five at evenly distributed timeframe of 16 seconds. A video is shown, which indicates when to initiate each movement as indicated by the program.
- The subject must perform again all ten movements with his/her left hand.
- For both steps (a) and (b), the number of events must be equal to 5 and must be supervised by an expert physiotherapist at all times.
- Since there are 8 electrodes for each event, each movement, each hand, and each subject, relevant electrodes must be selected using mutual information equations.
- In case the set of signals for a specific movement are unreadable or the subject did not perform the movement correctly, this section of the experiment must be repeated to ensure repeatability of the movements across all subjects.
- If the events for each movement are registered, the embedded noise must be removed. In this contribution, wavelet denoising was used.
- The filtered signals must be then normalized. It is possible to calculate cross recurrence quantification parameters to signals with different amplitudes as long as all the signals belong to the same system [51]. However, in this case study, the length of each signal is normalized for simplicity and to ensure repeatability.
- Once the signals were selected, filtered, and normalized, the recurrence parameters must be calculated.
- To ensure consistency, the parameters such as the norm, the time delay, and the embedded dimension are calculated as follows: dimension *m* = 6 using fixed nearest neighbors method, delay = 1 using average mutual information and Euclidean norm.
- Signals were processed using CRP tools such as Recurrence Rate, Determinism, Entropy, Laminarity, Trapping Time, and Trend must be calculated for the relevant signals for each movement and each hand for every subject unless a test was discarded as specified on step (c).
- Once the CRQ features were determined, the interpretation of the results must be carried out.

**Figure 5:**
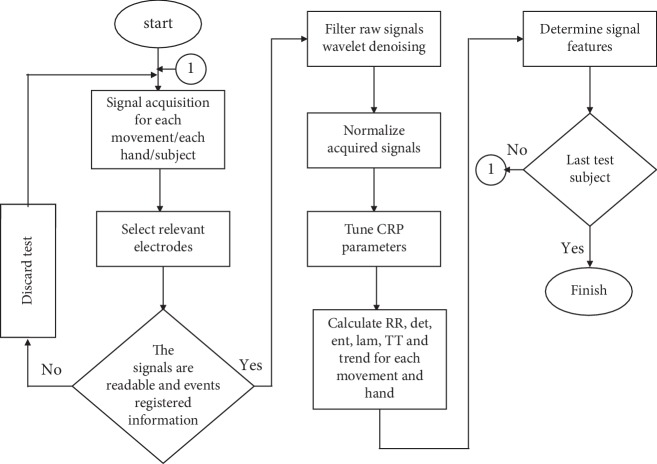
Methodology used in the present work.

The methodology described in the present section may be graphically shown in Figure 5.

**Figure 6:**
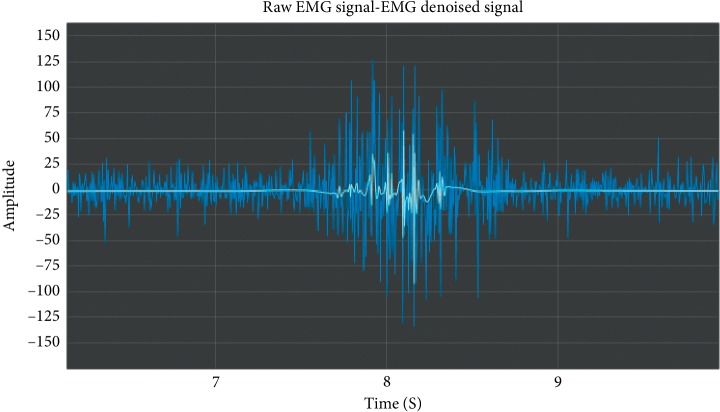
Example of a raw EMG signal event (dark blue) and its corresponding filtered signal by using Symlet 8 wavelet (in pale blue). Signal corresponding to wrist extension, right hand, subject 016.

As shown in the literature review of the present study, many authors have used a method to decompose or extract the necessary features before classifying the EMG signals. In this work, the raw signal filtering (as shown on Figure 5) is carried out using wavelet denoising. An example of a raw signal and its corresponding filtered signal using wavelet denoising (symlet 8 outperforms the other mother wavelts in this contribution) is shown on Figure 6.

**Figure 7:**
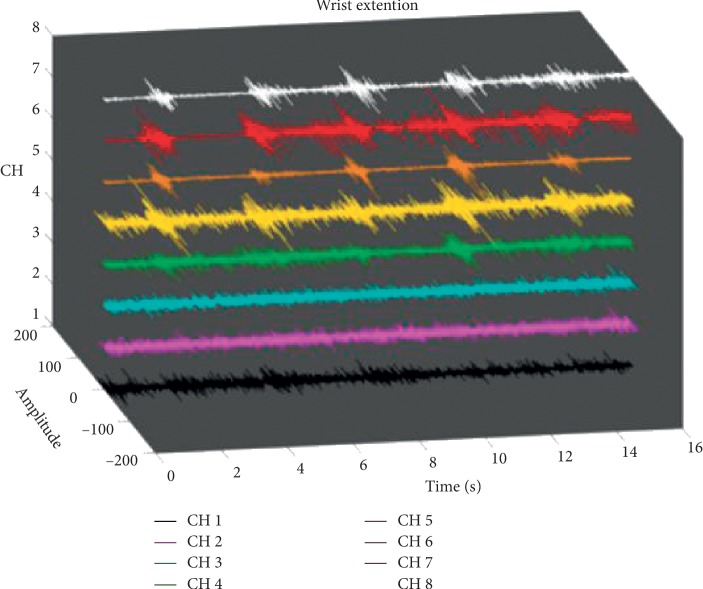
Example of a movement performed by test subject 016. The figure shows the time, the amplitude of the signal, and each of the recorded channels for the movement wrist extension (right hand).

Furthermore, a feature selection and reduction method to reduce the complexity and number of channels and finally cross recurrence plots (CRPs) to classify the movements, which may be a challenging task considering that each movement of each hand of each subject consists of 8 channels as seen on Figure 7.

## 4. Experimental Results

**Figure 8:**
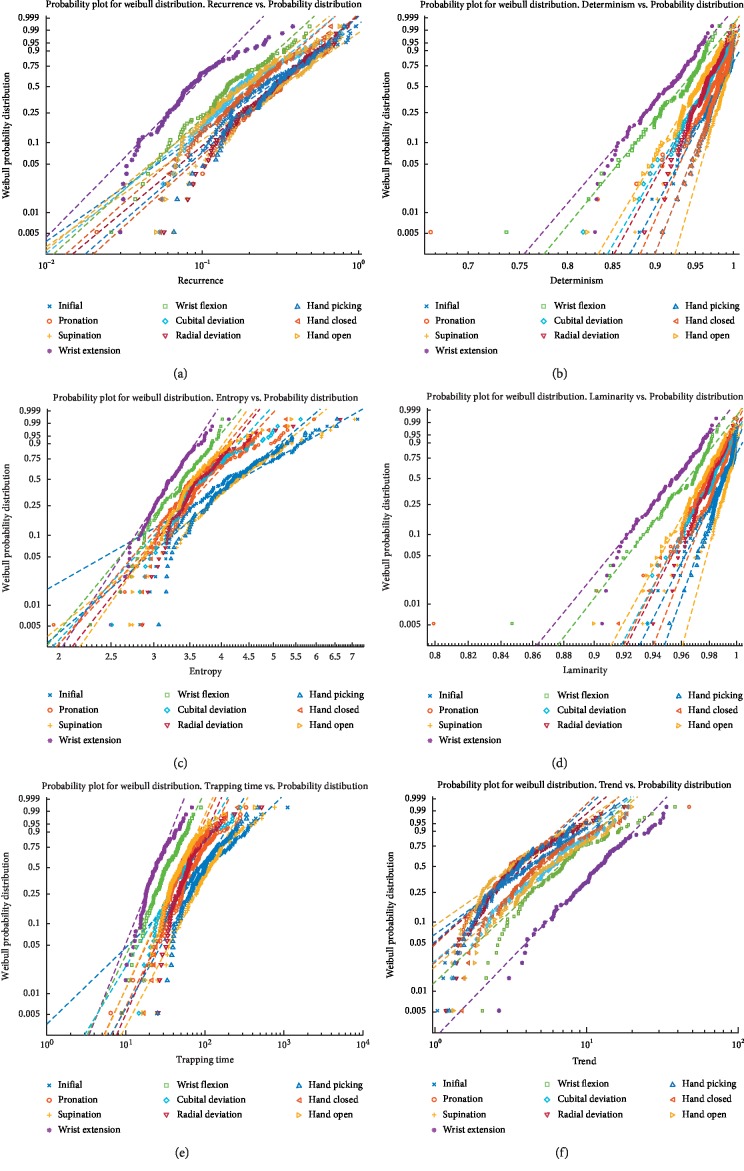
Results of RQA measures using the Weibull distribution. (a) Recurrence rate vs probability distribution, (b) Determinism vs probability distribution, (c) Entropy vs probability distribution, (d) Laminarity vs probability distribution, (e) Trapping Time vs probability distribution, and (f) Trend vs probability distribution.

As explained previously, the interpretation of this highly dimensional, nonlinear sEMG signals is often not easy to interpret. The results presented in this contribution were separated according to the six significant features in the cross recurrence quantification analysis explained in Section 2.4. A Weibull distribution was used to show the trend of each feature regarding each movement (Figure 8).

Figure 8(a) shows the recurrence rate for all tests for each movement. In this figure, it could be seen that there are some trends regarding the recurrence rate for each movement. For instance, wrist extension is shown considerably apart from the other movements, whilst movements such as radial deviation and hand picking are shown to be overlapped in this figure. Other movements, such as wrist flexion and hand open, among others, are well defined in its recurrence rate. Also, Figure 8(b) shows the determinism for all tests showing each movement. In this figure, Determinism has shown that each movement is differentiated from each other. This is specially so for wrist extension and wrist flexion. Likewise, Entropy shows a clear distribution for wrist extension, wrist flexion, and hand picking (Figure 8(c)). For Figure 8(d), Laminarity shows a clear distribution regarding wrist extension, wrist flexion, hand picking, and hand open, whereas Figure 8(e) shows also a characteristic distribution regarding wrist extension, cubital wrist deviation, and radial wrist deviation. Lastly, trend shows a characteristic wrist extension and wrist flexion trend.

To determine to which extent this proposed methodology could be used to classify the movements, sensitivity (SE), specificity (SP), and accuracy (AC) were calculated for each CRQA metric, which are extensively used for detection and classification metrics. This is done to avoid misinterpretation of the results, since the measure of the misdetection of movements must be considered. The misdetection is given by the false positive (FP) and false negative (FN), whereas a correctly detected signal is measured in terms of the true positive (TP) and true negative (TN). SE, SP, and AC are given by

(10) $$\begin{array}{c} \text{sensitivity}=\frac{\text{TP}}{\text{TP}+\text{FN}},\\ \text{specificity}=\frac{\text{TN}}{\text{TN}+\text{FP}},\\ \text{accuracy}=\frac{\text{TP}+\text{TN}}{\text{TN}+\text{TP}+\text{FN}+\text{FP}}. \end{array}$$

In this study, the quality metrics such as sensitivity, specificity, and accuracy were separated in the experiments carried out by each subject on each hand as shown in Tables 2–7. In these tables, each of the quality metrics was performed for each CRQA measure and each movement.

Table 2 shows the sensitivity, specificity, and accuracy of recurrence rate for each surface EMG movement performed in this experiment. In this table, it is shown that both the sensitivity and specificity for each experiment are considerable reliable for the experiments reaching 0.9786 for flexion, 0.9426 for extension, and 0.9364 for pronation, whereas the lowest is reached for hand picking with sensitivity of 0.7749 and specificity of 0.742. This seems to indicate that most movements are not susceptible to miscalculations of false negatives or false positives. In terms of accuracy, it is noticeable that the highest accuracy is also high, reaching 0.9433 for extension and 0.9372 for flexion, reaching the right hand 0.9688 and 0.9433, respectively, which seems to indicate a high classification precision. Likewise, it is noteworthy that most metrics show a higher quality metric for all movements using the right hand. This must be further investigated, but this seems to indicate that since most people who performed the experiment is right handed, they tend to perform the experiment more skillfully with the right hand, hence the difference in the results.

Table 3 shows the sensitivity, specificity, and accuracy of determinism for each movement performed in this experiment. In this table, is shown that also, the right hand shows slightly better results than the left hand. In this table, is shown than cubital wrist deviation shows the lowest sensitivity and specificity, which might be compensated by the sensitivity for this movement using recurrence rate as CRQA metric, which seems to indicate that the combination of all the features might result on a better rate. Also, eight of the movements show higher than 0.9 sensitivity and specificity for determinism which seems to indicate that this feature tends to avoid false positive and false negatives and give consistent results. The accuracy of determinism is also high for most metrics.

Table 4 shows the sensitivity, specificity, and accuracy of entropy for each movement performed in this experiment. In this table, the sensitivity, specificity, and accuracy of extension, flexion, and hand picking is high. Also, accuracy for most movements shows consistency which seems to indicate that the detection of true positives and true negatives is also constant. Also, these results seem to indicate that the entropy of the system, this is, the chaotic transitions, is detected with a reasonable accuracy by using this methodology.

Table 5 shows the sensitivity, specificity, and accuracy of laminarity for each movement performed in this experiment. In this table is shown that the laminar phases of most of the SEMG movements are also detected with moderate accuracy. In terms of accuracy, it is also shown that laminarity provides a robust window to detect TP and TN with a highest accuracy of 0.929 for right hand open, 0.9253 for right hand flexion, and 0.911 for extension. The lowest accuracy is for hand closed with 0.8388, which is not a poor value by any means.

Table 6 shows the sensitivity, specificity, and accuracy for trapping time for each SEMG movement. In this table is shown that most movements show consistency with the other CRQA features. Also, it is noteworthy that for trapping time, extension and flexion show also a high accuracy. The exception for this is hand closed where a sensitivity of 0.7938, specificity of 0.7821, and accuracy of 0.7964 are seen. This table also shows that most right-hand movements show a slightly higher SE, SP, and AC than its corresponding left hand movement, which is also fairly consistent with the results of the other CRQA metrics so far.

**Figure 9:**
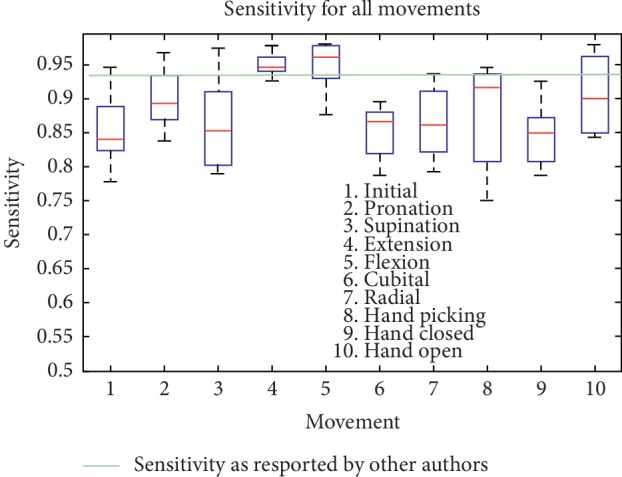
Sensitivity quality metric for each movement (every CRQA feature).

**Figure 10:**
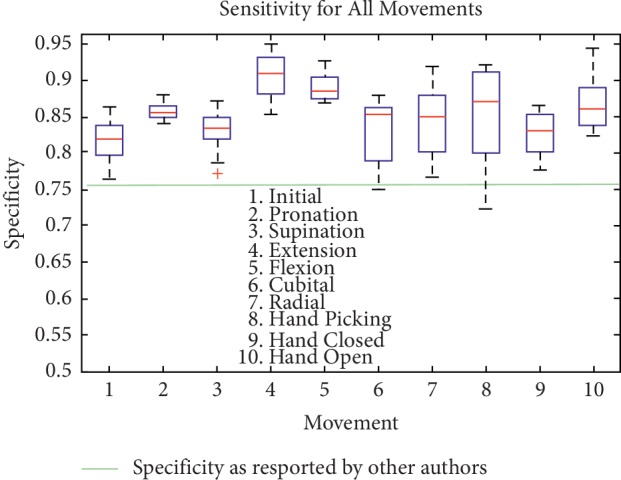
Specificity quality metric for each movement (every CRQA feature).

**Figure 11:**
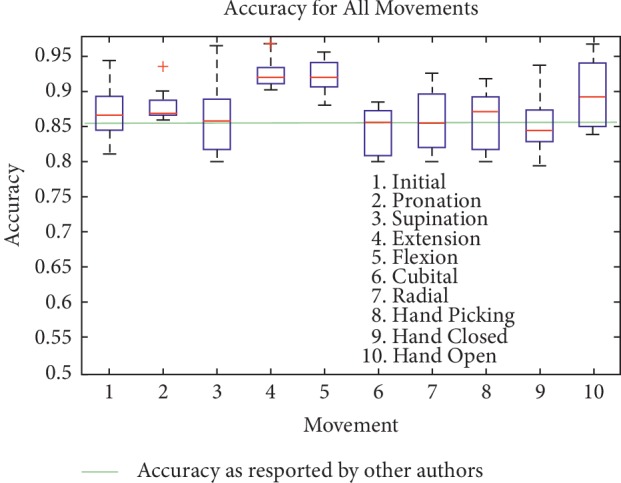
Accuracy quality metric for each movement (every CRQA feature).

Finally, Table 7 the quality metric SE, SP, and AC for CRQA trend for each SEMG movement. In this table, it is noteworthy that also for this feature, the results are considerably constant. To view the extent to which SE, SP, and AC are reasonably constant over all CRQA features, Figures 9–11 show the sensitivity, specificity, and accuracy for each movement, respectively.

In Figure 9 is shown the sensitivity metric applied to each CRQA feature for each movement. There is one box for each movement, which is indicated by the legend on the boxplot. Each box has lines at the lower quartile, median (red line across each box), and upper quartile values. The so-called whiskers are represented by a line extending from each end of each box to show the distribution of the rest of the data. In this figure, there are no outliers (normally represented by a red star) visible.

In Figure 9 is shown that most movements may be precisely detected and the number of false negatives is rather low. The median for all movements are 0.8392 for initial position, 08931 for pronation, 0.8516 for supination, 0.9468 for extension, 0.9609 for flexion, 0.8665 for cubital wrist deviation, 0.8607 for radial wrist deviation, 0.9157 for hand picking, 0.8482 for hand closed, and finally, 0.8997 for hand open, which seems to indicate that the detection is high using this methodology, and having the mean and percentiles fairly constant and the spread of the lower to upper percentile shows that the proposed method is consistent and robust. Both extension and flexion movements show a slightly higher sensitivity than the rest of the movements; the reason for this needs further investigation. However, it may be that these movements are less susceptible to have less false negatives and to be misdetected. Figure 9 also shows a green horizontal line, which represents the sensitivity as reported by other methodologies [36].

Figure 10 shows the specificity applied to every CRQA feature for each movement. In this case, specificity shows the extent to which there is a misdetection of a movement, that is, a movement is different in respect to the one that has been investigated (false negative). In this figure, the median seem also considerably steady, which seems to indicate the high capability of the methodology to detect both false positives (in sensitivity) as well as false negatives (for specificity, Figure 10). The only exception for this seems to be hand picking movement which shows a larger spread of the percentiles. Nevertheless, the median for this movement is shown to be high (0.8714). The median for the specificity of all movements as shown in Figure 10 is as follows: 0.8193 for initial position, 0.8546 for pronation, 0.8337 for supination, 0.9091 for extension (also the highest for specificity), 0.8852 for flexion, 0.8544 for cubital wrist deviation, 0.8504 for radial wrist extension, 0.8714 for hand picking as mentioned before, 0.8286 for hand closed, and 0.8597 for hand open. Figure 10 also shows the specificity obtained using a different machine learning methodology as reported by other authors (e.g., [36]).

It is noteworthy that for specificity as well as sensitivity, the highest values are for both flexion and extension. The muscles involved for both movements are the same; nevertheless, the movement is finished in an opposite direction, which shows that the proposed methodology may detect the phase transitions caused by the trajectory of the signals with high precision.

Figure 11 shows the accuracy for every CRQA feature for each movement. The median for this metric for each movement is as follows: 0.8668 for initial position, 0.8688 for pronation, 0.8581 for supination, 0.9202 for extension, 0.9196 for flexion, 0.8559 for cubital wrist deviation, 0.8553 for radial wrist deviation, 0.8719 for hand picking, 0.8441 for hand closed, and 0.8917 for hand open.

In this figure is also noteworthy that the highest accuracy is given by both flexion and extension movements, which seems to indicate robustness in the detection and the avoidance of misdetection of these movements. Also, hand picking movement that showed a large spread in the percentiles for both sensitivity and specificity is not as large with accuracy. This seems to indicate that, for most movements, the detection of true positives and true negatives is much higher, resulting in a higher accuracy value. Figure 11 also shows the mean accuracy (green horizontal line) as reported by other authors [36].

Comparing the results from the Tables 2–7 and Figures 9–11 is not an easy task. This is specially so because there are not many methodologies presented using the movements and the experimental setup as the authors made in the present study. However, studies such as the one presented by Nalband et al. [36] for knee-joint disorders may be a good indication on whether this methodology might be robust and consistent. In such a study, machine learning techniques such as random forest were presented, and the results were shown in terms of sensitivity, specificity, and accuracy as well.

In terms of specificity, Nalband et al. [36] showed results of 0.7568, 0.7838, and 0.8684. This showed that the proposed methodology in this study is able to detect with a slightly higher error rate. However, the muscles involved and the experimental setup are considerably different. In terms of accuracy, Nalband et al. [36] showed results of 0.8652, 0.8977, and 0.9101 which are fairly reasonable results in comparison with the results presented in this study. Lastly, the same authors (2016) showed sensitivity of 0.9423, 0.9615, and 0.9411 which is higher than the sensitivity presented in this study. However, as stated before, that does not mean that the methodology proposed is not correct or accurate, since there are many reasons as to why the sensitivity is slightly lower. Future work might lead to more experiments to determine whether sensitivity rate can be improved.

## 5. Conclusions and Future Work

The methodology proposed showed that it is feasible to detect movements using the methodology proposed in the present work, with an improvement upon other methods. Also, it was found that the left hand showed slightly lower sensitivity, specificity, and accuracy. Although, the difference is not considerably large, this may need further investigation. CRQA proves to be a reliable and robust tool to determine signals for SEMG even when the movements are casual as it was intended in this contribution. Some CRQA features prove to give better results than others, so the right tools must be selected in order to have an increase in accuracy.

Movements such as extension and flexion show a high sensitivity, specificity, and accuracy for most CRQA features, which is consistent with the robustness the authors try to achieve with the proposed methodology. Also, since extension and flexion are movements that exhibit an opposite trajectory from each other, the authors may conclude that CRQA features are able to detect the transition, dynamics, and trajectories of the movement, buried in the signal itself. Likewise, with cubital and radial wrist deviation, whereas the accuracy is lower in comparison with extension and flexion, most movements show a high precision in detecting the right signals and avoiding when a misdetection might occur.

Furthermore, it is noteworthy that most machine learning methods rely on the effectiveness of a preexisting model to describe the behavior of the data. In the case of the proposed methodology, no learner or model is needed beforehand and the classification may be carried out on real time without training of the data.

Lastly, this work shows that the proposed methodology may avoid false positive and false negative classifications with good accuracy, which is also a contribution of the present work.

For future work, it might be a good direction to include more experiments to determine whether sensitivity rate can be improved and the reason why the left hand showed a slightly lower detection quality metrics.

## Figures and Tables

**Table 1:** Age and gender distribution of the sEMG experiments.

Age	Women	Men
18–30	12	17
30–49	6	10
50–60	3	2
Total	21	29

**Table 2:** Sensitivity, specificity, and accuracy metrics for CRQA recurrence rate for each SEMG movement performed in this experiment.

Recurrence	Initial	Pronotion	Supination	Extension	Flexion	Cubital	Radial	Picking	Closed	Open
Sensitivity (SE)	**0.9064**	**0.9364**	**0.909**	**0.9426**	**0.9786**	**0.8611**	**0.8073**	**0.7749**	**0.8949**	**0.952**
SE left hand	0.8713	0.9113	0.8857	0.9252	0.977	0.8431	0.7932	0.7512	0.866	0.9362
SE right hand	0.9416	0.9616	0.9324	0.9601	0.9802	0.8732	0.8215	0.7987	0.9238	0.9678
Specificity (SP)	**0.8285**	**0.861**	**0.8389**	**0.862**	**0.8778**	**0.8319**	**0.7834**	**0.742**	**0.842**	**0.872**
SP left hand	0.793	0.8512	0.8339	0.8554	0.8713	0.8183	0.7664	0.7228	0.8183	0.8616
SP right hand	0.864	0.8708	0.844	0.8687	0.8843	0.8656	0.8004	0.7613	0.8657	0.8824
Accuracy (ACC)	**0.9152**	**0.8997**	**0.8773**	**0.9442**	**0.9372**	**0.8745**	**0.8251**	**0.8126**	**0.9076**	**0.9397**
ACC left hand	0.8874	0.8664	0.855	0.9196	0.9312	0.8718	0.8112	0.8006	0.8771	0.924
ACC right hand	0.9431	0.933	0.8996	0.9688	0.9433	0.8772	0.8391	0.8246	0.9382	0.9555

**Table 3:** Sensitivity, specificity, and accuracy metrics for CRQA determinism for each SEMG movement performed in this experiment.

Determinism	Initial	Pronotion	Supination	Extension	Flexion	Cubital	Radial	Picking	Closed	Open
Sensitivity (SE)	**0.9236**	**0.9341**	**0.9586**	**0.9479**	**0.9727**	**0.8047**	**0.9177**	**0.9062**	**0.8995**	**0.9733**
SE left hand	0.9012	0.9132	0.9441	0.9283	0.9681	0.7883	0.8991	0.8982	0.8116	0.969
SE right hand	0.9461	0.9551	0.9732	0.9676	0.9773	0.8212	0.9364	0.9143	0.9215	0.9776
Specificity (SP)	**0.8216**	**0.8646**	**0.8646**	**0.9773**	**0.8779**	**0.7558**	**0.8789**	**0.8482**	**0.8543**	**0.883**
SP left hand	0.8007	0.8498	0.8575	0.8633	0.8696	0.8613	0.8442	0.8451	0.8505	0.8713
SP right hand	0.8425	0.8794	0.8717	0.8914	0.8862	0.7504	0.8916	0.8514	0.8582	0.8947
Accuracy (ACC)	**0.9044**	**0.8835**	**0.9437**	**0.944**	**0.9464**	**0.8011**	**0.9055**	**0.8819**	**0.8749**	**0.906**
ACC left hand	0.8773	0.8673	0.923	0.9209	0.9371	0.8014	0.8848	0.8672	0.8686	0.9542
ACC right hand	0.9316	0.8997	0.9644	0.9672	0.9557	0.8009	0.9263	0.8967	0.8813	0.9668

**Table 4:** Sensitivity, specificity, and accuracy metrics for CRQA Entropy for each SEMG movement performed in this experiment.

Entropy	Initial	Pronotion	Supination	Extension	Flexion	Cubital	Radial	Picking	Closed	Open
Sensitivity (SE)	**0.7884**	**0.8877**	**0.7978**	**0.9602**	**0.9658**	**0.8728**	**0.8469**	**0.931**	**0.8071**	**0.8528**
SE left hand	0.7772	0.8761	0.7913	0.9441	0.9538	0.8662	0.8338	0.9172	0.8115	0.8447
SE right hand	0.7997	0.8994	0.8044	0.9163	0.9779	0.8794	0.8601	0.9449	0.8028	0.861
Specificity (SP)	**0.7764**	**0.8606**	**0.7797**	**0.9001**	**0.9001**	**0.863**	**0.8466**	**0.8925**	**0.8039**	**0.8255**
SP left hand	0.7643	0.8435	0.7724	0.8937	0.8866	0.8557	0.8296	0.8936	0.8003	0.8225
SP right hand	0.7885	0.8778	0.7871	0.9065	0.9137	0.8703	0.8637	0.8915	0.8076	0.8286
Accuracy (ACC)	**0.8169**	**0.8785**	**0.8068**	**0.9199**	**0.9284**	**0.8758**	**0.8611**	**0.912**	**0.8551**	**0.8616**
ACC left hand	0.8113	0.8661	0.8002	0.9032	0.9115	0.867	0.855	0.9057	0.8441	0.8559
ACC right hand	0.8226	0.891	0.8134	0.9366	0.9454	0.8846	0.8672	0.9184	0.8662	0.8673

**Table 5:** Sensitivity, specificity, and accuracy metrics for CRQA laminarity for each SEMG movement performed in this experiment.

Laminarity	Initial	Pronotion	Supination	Extension	Flexion	Cubital	Radial	Picking	Closed	Open
Sensitivity (SE)	**0.8492**	**0.877**	**0.8541**	**0.9499**	**0.9608**	**0.807**	**0.8703**	**0.932**	**0.86**	**0.9493**
SE left hand	0.8313	0.8671	0.8432	0.9432	0.9524	0.7997	0.8614	0.9338	0.8537	0.9436
SE right hand	0.8672	0.8869	0.8651	0.9567	0.9692	0.8143	0.8793	0.9302	0.8664	0.9551
Specificity (SP)	**0.8195**	**0.8561**	**0.8484**	**0.9181**	**0.8867**	**0.7894**	**0.8504**	**0.9199**	**0.8525**	**0.9387**
SP left hand	0.8164	0.8534	0.8401	0.9118	0.8762	0.7813	0.8433	0.9225	0.8488	0.9327
SP right hand	0.8226	0.8588	0.8567	0.9245	0.8973	0.7976	0.8576	0.9173	0.8562	0.9448
Accuracy (ACC)	**0.8886**	**0.8682**	**0.8528**	**0.9086**	**0.916**	**0.8092**	**0.8463**	**0.8661**	**0.8388**	**0.9226**
ACC left hand	0.8778	0.8662	0.8443	0.9063	0.9067	0.8015	0.8557	0.8557	0.8335	0.9162
ACC right hand	0.8995	0.8703	0.8613	0.911	0.9253	0.8169	0.8369	0.8766	0.8441	0.929

**Table 6:** Sensitivity, specificity, and accuracy metrics for CRQA trapping time for each SEMG movement performed in this experiment.

Trapping time	Initial	Pronotion	Supination	Extension	Flexion	Cubital	Radial	Picking	Closed	Open
Sensitivity (SE)	**0.8285**	**0.8689**	**0.8466**	**0.9513**	**0.8873**	**0.8889**	**0.9275**	**0.9395**	**0.7938**	**0.8479**
SE left hand	0.8218	0.9661	0.8332	0.9441	0.8764	0.8832	0.9213	0.9376	0.7883	0.8514
SE right hand	0.8352	0.8717	0.8601	0.9586	0.8993	0.8947	0.9337	0.9415	0.7993	0.8445
Specificity (SP)	**0.8169**	**0.8573**	**0.8281**	**0.942**	**0.8754**	**0.8695**	**0.9112**	**0.9044**	**0.7821**	**0.848**
SP left hand	0.8115	0.8558	0.8227	0.9339	0.8705	0.8615	0.9046	0.9062	0.7774	0.8413
SP right hand	0.8223	0.8589	0.8335	0.9502	0.8816	0.8776	0.9178	0.9127	0.7869	0.8548
Accuracy (ACC)	**0.8461**	**0.8804**	**0.8719**	**0.9169**	**0.881**	**0.87**	**0.908**	**0.8831**	**0.7964**	**0.8483**
ACC left hand	0.8444	0.8773	0.8668	0.9113	0.8817	0.8718	0.9071	0.8773	0.7952	0.8466
ACC right hand	0.8479	0.8835	0.877	0.9225	0.8804	0.8683	0.9089	0.8889	0.7976	0.8501

**Table 7:** Sensitivity, specificity, and accuracy metrics for CRQA trend for each SEMG movement performed in this experiment.

Trend	Initial	Pronotion	Supination	Extension	Flexion	Cubital	Radial	Picking	Closed	Open
Sensitivity (SE)	**0.8344**	**0.8405**	**0.7942**	**0.9434**	**.09299**	**0.8702**	**0.8109**	**0.8068**	**0.8329**	**0.8536**
SE left hand	0.8256	0.8432	0.7998	0.9372	0.9263	0.8671	0.8173	0.8032	0.8226	0.8441
SE right hand	0.8432	0.8379	0.7887	0.9496	0.9355	0.8734	0.8045	0.8104	0.8427	0.8632
Specificity (SP)	**0.8443**	**0.8434**	**0.8226**	**0.9309**	**0.9196**	**0.8542**	**0.8392**	**0.8009**	**0.8286**	**0.8457**
SP left hand	0.8327	0.84	0.8183	0.9284	0.9116	0.8563	0.8004	0.7987	0.8203	0.8337
SP right hand	0.8559	0.8468	0.8269	0.9335	0.9277	0.8532	0.8012	0.8031	0.8369	0.8578
Accuracy (ACC)	**0.8517**	**0.8626**	**0.8174**	**0.9242**	**0.9107**	**0.8392**	**0.801**	**0.8042**	**0.8278**	**0.8508**
ACC left hand	0.8471	0.8667	0.8115	0.9178	0.9068	0.8337	0.7994	0.8005	0.8342	0.8403
ACC right hand	0.8563	0.8585	0.8234	0.9307	0.914	0.8448	0.8026	0.8079	0.8214	0.8615

## Data Availability

The data are stored in the Mendeley database. The database can be downloaded in the Mendeley database: https://data.mendeley.com/datasets/p77jn92bzg/1.
